# Missing data in randomized controlled trials testing palliative interventions pose a significant risk of bias and loss of power: a systematic review and meta-analyses

**DOI:** 10.1016/j.jclinepi.2015.12.003

**Published:** 2016-06

**Authors:** Jamilla A. Hussain, Ian R. White, Dean Langan, Miriam J. Johnson, David C. Currow, David J. Torgerson, Martin Bland

**Affiliations:** a Hull York Medical School, University of York, Heslington, York YO10 5DD, UK; b MRC Biostatistics Unit, Cambridge Institute for Public Health, Forvie Site, Robinson Way, Cambridge CB2 0SR, UK; c Centre for Reviews and Dissemination, University of York, Heslington, York YO10 5DD, UK; d SEDA, Hertford Building, University of Hull, Hull HU6 7RX, UK; e Palliative and Supportive Services, Flinders University, 216 Daws Road, Daw Park, South Australia 5041, Australia; f York Trials Unit, University of York, Heslington, York YO10 5DD, UK; g Health Sciences Department, University of York, Heslington, York YO10 5DD, UK

**Keywords:** Systematic review, Meta-analysis, Palliative care, Randomized controlled trials, Missing data, Differential mortality

## Abstract

**Objectives:**

To assess the risk posed by missing data (MD) to the power and validity of trials evaluating palliative interventions.

**Study Design and Setting:**

A systematic review of MD in published randomized controlled trials (RCTs) of palliative interventions in participants with life-limiting illnesses was conducted, and random-effects meta-analyses and metaregression were performed. CENTRAL, MEDLINE, and EMBASE (2009–2014) were searched with no language restrictions.

**Results:**

One hundred and eight RCTs representing 15,560 patients were included. The weighted estimate for MD at the primary endpoint was 23.1% (95% confidence interval [CI] 19.3, 27.4). Larger MD proportions were associated with increasing numbers of questions/tests requested (odds ratio [OR], 1.19; 95% CI 1.05, 1.35) and with longer study duration (OR, 1.09; 95% CI 1.02, 1.17). Meta-analysis found evidence of differential rates of MD between trial arms, which varied in direction (OR, 1.04; 95% CI 0.90, 1.20; *I*^2^ 35.9, *P* = 0.001). Despite randomization, MD in the intervention arms (vs. control) were more likely to be attributed to disease progression unrelated to the intervention (OR, 1.31; 95% CI 1.02, 1.69). This was not the case for MD due to death (OR, 0.92; 95% CI 0.78, 1.08).

**Conclusion:**

The overall proportion and differential rates and reasons for MD reduce the power and potentially introduce bias to palliative care trials.

**What is new?**

• On average nearly a quarter of primary endpoint data are reported as missing in trials evaluating palliative interventions in participants with advanced disease (95% prediction interval 11.8, 67.7).
• Both increasing numbers of questions asked and/or tests requested and study duration were associated with larger proportions of missing data (MD).
• There was evidence of differential rates and reasons for MD between the intervention and control arms, which may introduces bias.
• MD in the intervention arm were more likely to be attributed to disease progression unrelated to the intervention, compared to the control arm. This indicates that there may be systematic misclassification of the reason for MD.

## 1 Introduction

Missing data (MD) are defined as observations that would be meaningful to analysis and are intended to be made, but for some reason are not [1]. There are two types of MD: *unit*, where no data are provided from a unit, for example, participant; and *item*, where particular items of data are not provided [2]. These can occur intermittently or in a monotone pattern when no further data are provided. Such MD reduce the power, precision, and, if certain groups of participants (ie, those who have a poor performance status) are missing, external validity of trial findings [3]. Furthermore, MD can pose a major threat to the internal validity of trial results [3]. This can occur particularly if there are significant differences between trial arms with regards to: (1) rates of MD, (2) the baseline characteristics of those who do not provide data, or (3) the reasons data are missing. Such differences indicate that the trial arms are no longer comparable for known and unknown factors, and therefore, the scientific benefits of randomization are compromised.

Trials of palliative interventions in participants with advanced life-limiting illnesses present an important case with regards to MD. Large amounts of MD due to death and disease progression unrelated to the intervention are expected in this population and do not necessarily reflect poor trial design and conduct [4], [5]. These MD will, however, reduce the power of the study to detect a true effect [3], [5] and potentially introduce bias if missingness is associated with treatment allocation. It is therefore important that trials are designed to minimize the extent of MD as much as possible [3], [4], [6] and that an evidence-based estimate of the proportion of MD is used to adjust the sample size for MD. A review of participant-level data from 18 symptom control palliative oncology studies from one center found the average proportion of attrition by the primary endpoint was 26% (95% confidence interval [CI] 23, 28%) [7]. However, it is unknown how this finding relates to a broader enquiry encompassing single site and multisite trials, led by different research centers, which evaluate a range of palliative interventions in participants with malignant and nonmalignant disease.

Inflation of the sample size to accommodate expected MD will improve the power of a study; however, it will not account for the bias caused by differential rates and reasons for MD [3]. It is essential therefore to understand which study design and participant factors are associated with both the overall amount and differential rates of MD and consequently how these can be minimized. Palliative care encompasses a wide variety of life-limiting illnesses and a broad range of pharmacological, psychological, and spiritual interventions. Therefore, it is expected that the rates of MD will vary considerably in this population, which will enable factors associated with MD to be assessed.

This systematic review and meta-analyses assessed the risk MD poses to trials testing palliative interventions. The specific aims of the review were to determine (1) the proportion of MD by the primary endpoint across a representative range of published palliative care trials; (2) trial design and participant factors associated with the overall proportion of MD at the primary end-point; (3) whether there is evidence of differential rates of MD and which study design factors are associated with this; (4) whether the baseline characteristics and reasons for MD differ between trial arms, thus potentially introducing bias.

## 2 Materials and methods

### 2.1 Eligibility criteria

Eligible studies were randomized controlled trials (RCTs) published between January 2009 and April 2014, that included adult *participants* with an advanced, progressive, life-limiting illness, with no possibility of remission [8]. Trials testing a palliative *intervention* where the primary aim is to improve quality of life, rather than modify the disease process or improve survival, were included. The nature of the intervention was not restricted, that is, trials testing pharmacological, surgical, psychospiritual, and communication skills interventions were eligible. Any *comparators* that were palliative in nature, standard care, or placebo were included. This review was limited to trials with patient-reported or patient-dependent primary *outcomes.* If the primary outcome was the proportion of MD, such studies were excluded. If the authors stated that the trial arms were established by random allocation, that is, the term “random(ized)” was used, then the *study* was considered to be an RCT and included [9]. A 5-year period was chosen to capture current practice and also overlapped with the publication of the CONSORT 2010 statement [10] which included clearer guidance on intention-to-treat (ITT) analysis and the implications of MD than previous statements. No language restrictions were applied.

### 2.2 Search strategy

An information specialist with the Pain, Palliative, and Supportive Care Group (PaPaS) [11] at the Cochrane Collaboration helped to formulate and conduct the search. The electronic databases searched to identify studies were CENTRAL (January 2009 to April 2014), OVID MEDLINE (January 2009 to April 2014), and EMBASE (January 2009 to April 2014). The search strategy combined a modified version of the Cochrane PaPaS palliative care search strategy [12] and the sensitivity maximizing Cochrane Highly Sensitive Search Strategy for identifying RCTs in MEDLINE [9] (see Appendix A at www.jclinepi.com). Authors of studies were not contacted for further studies because the aim was to provide a representative sample rather than to be exhaustive. To gain a representative sample, a computer-generated random sequence was used to select successive random samples of 100 studies, which were then screened until the *a priori* sample size of over 100 trials was reached.

### 2.3 Study selection and data extraction

Two reviewers independently and then in pairs conducted the screening, selection, and data extraction. One has a background in palliative and health care research (J.A.H.) and the other in statistics and clinical trials (D.L.), thus representing both content and methodological expertise [13]. If no agreement could be reached, an arbitrator was to be consulted. Agreement was measured using kappa statistics for categorical variables and intraclass correlation coefficient (ICC) for continuous variables. If there was insufficient information to make a decision about inclusion, authors were contacted via email and ResearchGate [14]. Studies published in more than one article were combined into one study.

### 2.4 Outcomes

In each study, the outcomes were (1) the proportion of MD at the primary endpoint (analyzed as log odds), for repeated measures where the time of the primary endpoint was not clearly specified, the final observation was taken as the primary endpoint; (2) differential rates of MD, defined as the log odds ratio (OR) of MD in the intervention arm compared to the control arm; (3) whether there were statistically significant differences in baseline characteristics between the intervention and control arms; (iv) differential reasons for MD, defined as the log ORs of missing due to death, missing due to disease progression, and missing due to toxicity, for the intervention arm compared to the control arm. MD in this review included data censored due to death and MD that could have been collected but was not. If there were more than one intervention arm, these were combined to represent all intervention arms; therefore, the participants in the control arm were not used to provide information on more than one effect size, thus ensuring the information for the different estimates was independent [15].

### 2.5 Analysis

The DerSimonian and Laird random-effects model (REM) meta-analysis was used for the proportion of MD and differential rates and reasons for MD [16]. The Cochran Q (χ^2^) test and the *I*^2^ statistic were used to analyze statistical heterogeneity [17]. Cochran Q test has low power when there are few studies or when included trials have a small sample size; therefore, *a priori* a *P*-value ≤ 0.10 was considered to be statistically significant [17], [18]. The potential sources of heterogeneity were prespecified as following:

A. Proportion of MD at the primary endpoint:
  • “items of data requested”: this is the total number of individual questions and tests the participants were required to complete during the course of the trial
  • time to the primary endpoint (days)
  • total number of outcome measures and measurement frequency
  • nature and design of the trial; sample size; multisite/single site; funding source
  • nature of the outcome, intervention, data collection method, and setting
  • exclusion of participants who are older, with a poorer performance status or advanced disease
  • use of specified methods to minimize MD
  • trial participants' average age, performance status, and underlying disease
  • risk of bias due to random sequence generation, allocation concealment, and blinding
B. Differential rates of MD
  • nature of the intervention and control
  • blinding of participants
  • items of data requested
  • time to the primary endpoint (days).

All potential sources of heterogeneity were assessed in a univariate metaregression analysis fitted by the residual maximum likelihood method. The sources of heterogeneity were further tested using the univariate and multivariate Monte Carlo permutation test, which takes account of multiple testing (1,000 permutations per analysis) [19]. The assumption of normality of the random effect for the metaregression model was assessed using a normal probability plot of the standardized predicted random effects, which demonstrated that the assumption was adequate. Analyses were conducted using Comprehensive Meta-Analysis v2.2 and STATA v13. For all analyses, except those specified, the level of statistical significance was set at 5%.

## 3 Results

### 3.1 Study selection

**Fig. 1:**
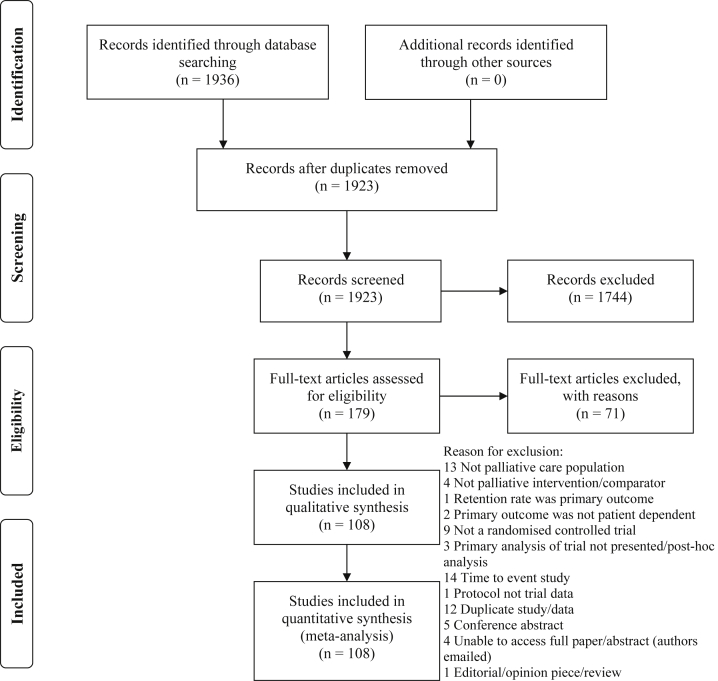
PRISMA study flow diagram.

Of the 1,923 titles and abstracts screened, 1,744 did not meet the inclusion criteria. Full-text articles of 179 studies were assessed for eligibility of which 108 were included in the final analysis (Figure 1) and represented data from 15,560 randomized patient participants. For four articles, full-texts were not available from authors or local and national libraries [20], [21], [22], [23]; these are listed as “potentially relevant studies” (see Appendix B at www.jclinepi.com). Interrater agreement for categorical and continuous variables ranged from kappa statistic 0.64–1.0 and ICC 0.70–0.95; consultation with an arbitrator was not required.

### 3.2 Study characteristics

The demographics of the included studies and participants are presented in Table 1. Most were parallel trials (87%) and conducted in Europe and North America (73.1%). The mean age was 64 years, and average Eastern Cooperative Oncology Group performance status was 2 (defined as “ambulatory and capable of self-care but unable to carry out any work activities. Up and about for >50% of waking hours” [24]). The median individual number of questions asked/test requested was 106 (interquartile range [IQR], 37–231) and time to the primary endpoint was 28 days (IQR, 7–84).

**Table 1:** Demographics of included studies and participants

Trial demographics	Number (%)	Participant demographics	Frequency or average (%)
Study design		Number randomized	Median 68 IQR 36–129
Parallel trials	94 (87)	Age of intervention group	Mean 64.5, SD 8.5 Range 30–85
Crossover trials	10 (9.3)	Age of control group	Mean 64.3, SD 8.5 Range 30–86
Cluster trials	4 (3.7)	Life-limiting condition	
Nature of trial		Malignant disease	80 (74)
Feasibility	8 (7.4)	Nonmalignant disease	6 (5.6)
Pilot	15 (13.9)		
Phase 2	12 (11.1)		
Phase 3	7 (6.5)	Mixed	21 (19.4)
RCT^a^	66 (61.1)	Not reported	1 (0.9)
Multicenter or single center		Performance status (ECOG)	
Single	63 (58.3)	0	1 (0.9)
Multiple	44 (40.7)	1	13 (12.0)
Unclear	1 (0.9)	2	18 (16.7)
Number of centers in multicentre trials	Median 3, range 2–27	3	12 (11.1)
Continents		4	5 (4.6)
Europe	41 (38.0)	Others: 0–2^b^	1 (0.9)
North America	38 (35.2)	0–3^b^	2 (1.9)
Asia	18 (16.7)	KPS >50	1 (0.9)
Australia	6 (5.6)	Not reported	55 (50.9)
Africa	2 (1.9)	Median survival	Median 118 days IQR 67–192
>1 country	3 (2.8)	Not reported	73 (67.6)

*Abbreviations*: IQR, interquartile range; RCT, randomized controlled trial; ECOG, Eastern Cooperative Oncology Group; KPS, Karnofsky performance status.

a Allocation explicitly described as a variant of the term “random” but not described as a feasibility, pilot, phase 2 or phase 3 trial.

b Performance status presented as a range.

### 3.3 Proportion of MD on the primary endpoint

**Fig. 2:**
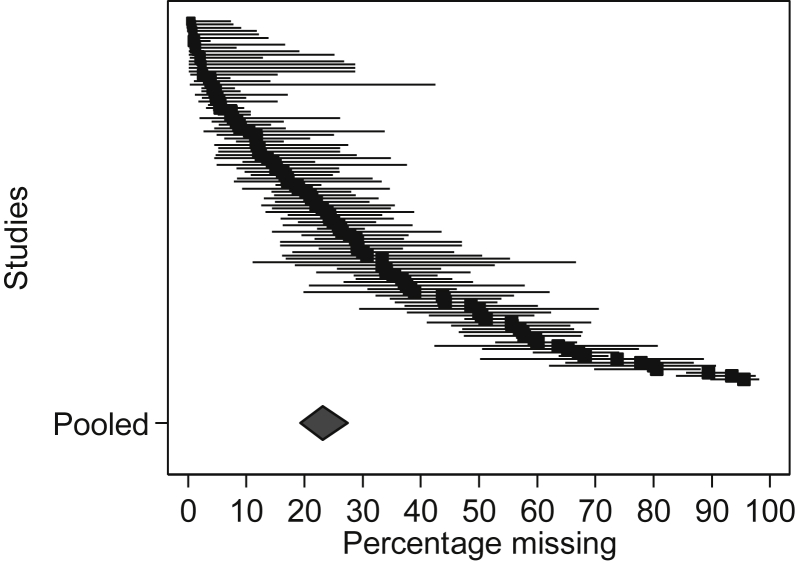
Forest plot of the reported proportion of missing data at the primary endpoint.

The weighted summary estimate for the proportion of MD on the primary endpoint was 23.1% (95% CI 19.3, 27.4; Figure 2). Fifty-six trials (51.9%) had greater than 20% MD on the primary endpoint. The majority of trials reported MD due to attrition only; item-level MD were only partially reported in 14 trials (13%). Heterogeneity was very large with *I*^2^ = 96.0% and *P* < 0.001. Tau^2^ was 1.2, which yields a 95% prediction interval of approximately 11.8 to 67.7, meaning the true proportion of MD in the next study is expected to fall anywhere between 11.8% and 67.7%. This large range reflects the heterogeneity between studies and the error in estimating the mean (which is minimized with the large sample size).

The high heterogeneity enabled an exploration of the relationship between the proportion of MD and study-level covariates using metaregression analysis (see Appendix C at www.jclinepi.com for the results of all covariates tested). Four variables had a statistically significant association with the proportion of MD: (1) items of data requested (OR, 1.27; 95% CI 1.13, 1.42, per doubling), (2) time to the primary endpoint (OR, 1.13; 95% CI 1.06, 1.21, per doubling), (3) type of outcome (with most MD for quality of life outcomes), and (4) whether participants were excluded based on their: age/performance status/extent of disease (OR, 1.65; 95% CI 1.08, 2.52; Table 2).

**Table 2:** Univariate and multivariate metaregression for the odds ratio of missing data at the primary endpoint

Type of analysis	Covariates	Odds ratio	*P*-value	Confidence intervals	Adjusted *P*-value^a^
Univariate regression	Items of data requested (per number of items doubling)	1.27	<0.001	1.13–1.42	<0.001
	Time to primary endpoint (per number of days doubling)	1.13	0.001	1.06–1.21	<0.001
	Type of outcome		0.02		—b
	Symptom^c^	1.00	—	—	
	Psychospiritual	1.14	0.7	0.61–2.16	
	Quality of life	1.91	0.04	1.02–3.57	
	Other	0.60	0.06	0.36–1.01	
	Exclude patients based on age/performance status/extent of disease	1.65	0.02	1.08–2.52	0.03
Multivariate regression	Items of data requested (per doubling number of items)	1.19	0.007	1.05–1.35	0.02
	Time to primary endpoint (per doubling number of days)	1.09	0.02	1.02–1.17	0.008

Multivariate model *P* < 0.001, adjusted *R*^2^ 21.0%, *I*^2^ residual = 95.4%, Tau^2^ 0.6.

a Adjusted for multiple testing (Monte Carlo permutation test).

b Not possible to determine.

c Reference category.

All four variables were assessed in a multivariate metaregression, and only (1) total items of data requested (*P* = 0.007) and (2) time to primary endpoint (*P* = 0.02) had a statistically significant association with the proportion of MD, whereas variables (3) type of outcome and (4) exclusion based on age/performance status/extent of disease appeared to lose statistical significance because of their association with the items of data requested and time to primary endpoint (data not shown). All results were confirmed by the Monte Carlo permutation test used to adjust for multiple testing (Table 2). The adjusted *R*^2^ was 21.0%; however, the residual *I*^2^ remained high at 95.4%. These results demonstrate that the odds of MD increased as the number of items of data requested and the number of days to the primary endpoint increased (Table 2). For the items of data requested, when the time to primary endpoint is constant, if the number of individual questions/tests requested is doubled, the MD OR increased by 1.19. For the time to the primary endpoint, when the number of questions/tests are constant, if the time to the primary endpoint doubles, the MD OR increased by 1.09.

### 3.4 Differential rates of MD

**Fig. 3:**
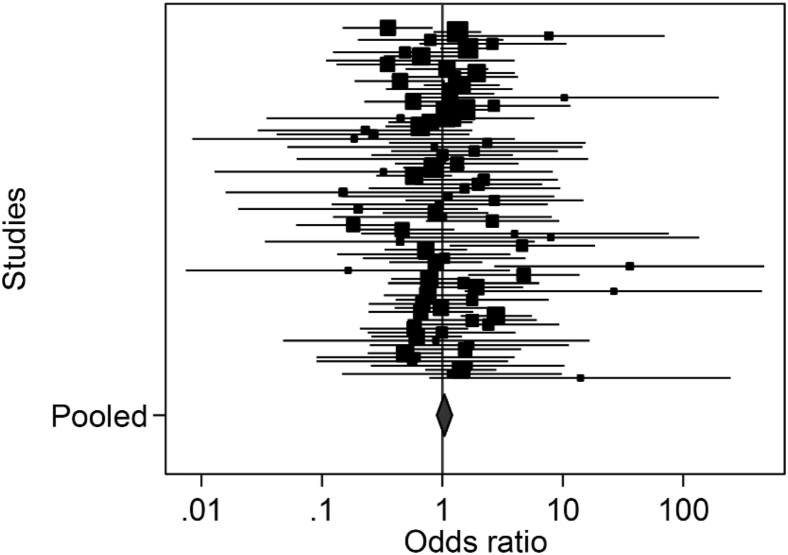
Forest plot of the reported differential proportion of missing data in the intervention compared to the control arm.

The median proportion of MD by the primary endpoint in the intervention arm was 21.6% (IQR, 6.5–45.1%) and in the control arm 20.0% (IQR, 3.8–41.8%). Meta-analysis of the log OR of the proportion of MD in the intervention compared to the control arm, resulted in an overall OR of 1.04 (95% CI 0.90, 1.20) with highly significant statistical heterogeneity (*I*^2^ 35.9, *P* = 0.001; Figure 3). This demonstrates that there is evidence of differential rates of MD between the trial arms, which varies in direction across trials.

The univariate and multivariate metaregression demonstrated there was insufficient evidence of a significant association between the differential rate of MD between trial arms and any of the prespecified factors tested: (1) nature of the intervention (*P* = 0.7), (2) nature of control (*P* = 0.2), (3) whether participants were blinded (*P* = 0.09), (4) items of data requested (*P* = 0.4), and (5) time to the primary endpoint (*P* = 0.4; see Appendix D at www.jclinepi.com).

### 3.5 Differential reasons for MD

Even if the proportion of MD in the trial arms is the same, if different types of people have MD in the different arms, this has the potential to introduce bias. No trial report reported a comparison of the baseline characteristics of those who withdrew from the intervention and control arms. There was no evidence of a difference in the proportion of MD due to death in the intervention arms compared to the control arms, across the trials (OR, 0.92; 95% CI 0.78, 1.08; Table 3). The heterogeneity in the trials was low, and there was no evidence of statistically significant variability between trials (*I*^2^ = 0.0, *P* = 0.6).

**Table 3:** Meta-analysis comparing the reasons for missing data in the intervention compared to the control trial arms

Reason for missing data	Death	Disease progression	Adverse effects
Number of trials	57	39	15
Point estimate	0.92	1.31	2.35
Lower limit	0.78	1.02	1.44
Upper limit	1.08	1.69	3.86
Cochran Q	51.8	26.3	14.7
DF	56	38	14
*P*-value	0.6	0.9	0.4
*I*^2^	0.0	0.0	5.0
Tau^2^	0.0	0.0	0.05

Abbreviation: DF, Degrees of freedom.

The estimate of the OR of those who had MD due to disease progression unrelated to the intervention was 1.31 (95% CI 1.02, 1.69; Table 3). This suggests that those in the intervention arm were significantly more likely to have MD that were attributed to disease progression, compared to those in the control arm. The *I*^2^ was 0.0 with no evidence of statistically significant heterogeneity (*P* = 0.9).

The number of trials that reported intervention related adverse effects (including toxicity) in the intervention and control arm was smaller than for the other categories (*n* = 15). The estimate of the OR of those who had MD attributed to adverse effects was 2.35 (95% CI 1.44, 3.86) with little heterogeneity between trials (*I*^2^ 5.0%, *P* = 0.4; Table 3). This indicates, as anticipated, that significantly more participants had MD due to treatment-related adverse effects in the intervention arm compared to the control arm.

## 4 Discussion

The development of a robust evidence base for individuals with advanced life-limiting illnesses that require palliative interventions is a key priority for patients, clinicians, and policy makers [25], [26]. This systematic review and meta-analyses demonstrates that on average, nearly a quarter of data are missing by the primary endpoint in trials evaluating palliative interventions in participants with advanced disease. This reduces the power and precision of trial results if not taken into account in the original power calculation and moreover can introduce systematic error through differential rates and reasons for MD. Such potentially biased results may have significant consequences for patient care subsequently informed by this evidence.

Although any level of MD is a potential risk to the internal and external validity of a trial, levels >20% are considered to pose a significant risk [27]. In this review, the weighted summary estimate for the proportion of MD by the primary endpoint was 23.1% (95% CI 19.3, 27.4%), similar to the proportion estimated in a review of 18 palliative-oncology trials [7]. As the trials were significantly heterogeneous, compared to the 95% CI, the 95% prediction interval (11.8, 67.7) more accurately describes the full uncertainty around the summary estimate in a way that acknowledges heterogeneity and can be applied in sample size calculations in future trials [28].

Over 50% of trials had >20% of data missing for the primary endpoint. This is much larger than the proportion calculated in three systematic reviews of trials published in general medical journals, which found the median proportion of MD to be 6% [29], 9% [30], and 10% [31]. The proportion reported in this review is also likely to be an underestimate as all but 14 trials only reported MD due to attrition (unit level); item-level MD were not reported systematically.

Our review assessed the burden of the trial in terms of the number of individual questions asked/tests requested, total number of outcomes, frequency of outcome measurement, method and setting of data collection, type of intervention and outcome, and duration. Only duration and the number of individual items of data collected had a statistically significant association with the proportion of MD. A review of palliative oncology trials also found duration to be associated with attrition at the primary endpoint (*P* = 0.04) and the end of the study (*P* = 0.01) [7]. They also found trials set in outpatients departments were significantly more likely than inpatients to have attrition (*P* = 0.05) [7]. Our large systematic review however did not find sufficient evidence of an association between the setting of the trial and MD (see Appendix C at www.jclinepi.com). Both the duration to the primary endpoint and number of individual items of data requested must be considered in the design of a trial, and if the research question and science necessitates that they cannot be minimized, adequate resources must be provided to ensure participants are supported to provide the outcome data. Together the two factors only explained 21% of the variance, which suggests other mediating factors need exploration.

There was evidence of differential rates of MD between intervention and control arms, which varied in direction. This suggests that there is a significant risk that the MD in these trials may introduce bias, and the direction of bias varies. Other reviews of differential rates of MD across a range of health care disciplines have reported mixed results [32], [33], [34], [35], [36], [37], with half reporting no evidence of differential attrition [32], [33], [34]. There was insufficient evidence that any of the prespecified covariates explained the heterogeneity, although the risk of bias associated with participant blinding was approaching statistical significance at the 5% level (*P* = 0.09). Crutzen et al. [35] in their review of health behavior change trials also did not find any explanatory variables to be significantly associated with the rate of differential attrition. However, an RCT of a psychosocial intervention on well-being after colorectal cancer surgery did find the probability of nonresponse decreased with increasing anxiety in the intervention group but increased with increasing anxiety in the control group [38]. These findings indicate that further exploration of factors that predict differential rates of MD is required.

Although the imbalances in the differential rates of overall MD varied across trials, imbalances for specific reasons for MD were found to be homogeneous. There was no evidence that the proportion of participants who died in the intervention compared to the control arms was significantly different after randomization. This is expected in RCTs testing interventions that are palliative and do not aim to improve survival, in a population where death unrelated to the intervention is expected. However, this was not the case for MD due to disease progression unrelated to the intervention (OR, 1.31; 95% CI 1.02, 1.69). In a population where disease progression unrelated to the intervention is expected, this is a surprising finding, as we would expect that through randomization, on average, both groups would be balanced in this regard. Some of the interventions may have had a survival advantage as a secondary gain, but the results indicate that more of the participants in the intervention arms withdrew because of disease progression. In the context of an adequately randomized trial, this can only be explained by a postrandomization effect and indicates strongly that there is systematic misclassification of the reasons for MD. A possible explanation is that intervention-related adverse effects are misattributed to disease progression unrelated to the intervention, thus underestimating the harm of interventions.

### 4.1 Limitations

Palliative care is an evolving, diffuse field, which spans multiple subject areas [39], with research published in specialist palliative care and general medical journals. The identification of relevant literature therefore is problematic, and eligible trials may have been missed. To address this, we used previously validated search strategies for both palliative care and RCTs and accessed the support of an information specialist. Four [40], [41], [42], [43] potentially relevant reports were not included as the full text could not be retrieved from local and national libraries or authors. As with any systematic review that only includes published research, there is a risk of publication bias, multiple publication bias, and reference bias [44]. The inclusion of published trials will also likely present an overoptimistic picture of the impact of MD in palliative care trials. Furthermore, we included data censored because of death in our definition of MD; however, this presents a different issue to MD in those alive [45], [46]. However, as all incomplete data impacts the interpretation of trial results, in this exploratory review *a priori* it was considered important for this to be quantified and assessed.

## 5 Conclusions

The average proportion of MD by the primary endpoint in palliative care RCTs is large and presents a significant risk to the power, precision, and generalizability of trial results. The minimization of MD is therefore essential, and this systematic review indicates that both the trial burden and duration need to be considered in trial design and sample size adjustment for MD. Further research is required to generate a theoretical framework to explain why MD occurs in these trials and in particular why differential rates of MD occur. This will help inform researchers on how to best reduce MD that is modifiable in this population as recommended in MD guidance [3], [4], [47]. Such marginal gains could have a significant impact on trial validity [48]. No trials compared the baseline characteristics of participants who had MD, but this information is important in the assessment of the risk of bias and should routinely be reported. Further research is also required into how the reasons for MD are assessed and documented, especially when differentiating MD due to disease progression and adverse effects in this population.

## Supplementary data

Appendices A–D
