# Supplementary material for: Missing data in randomized controlled trials testing palliative interventions pose a significant risk of bias and loss of power: a systematic review and meta-analyses
Source: J Clin Epidemiol. 2016 Jun;74:57–65. doi: 10.1016/j.jclinepi.2015.12.003 (PMC4910872; doi:10.1016/j.jclinepi.2015.12.003)
Supplement: Appendices A–D [file mmc1.docx]

**Appendix A: Search strategy**

The search terms and strategy used for the palliative care literature (displayed for the OVID Medline database) were as follows:

MESH TERMS are in uppercase

#1 PALLIATIVE CARE

#2 Exp TERMINAL-CARE

#3 TERMINALLY-ILL

#4 ATTITUDE-TO-DEATH

#5 Exp BEREAVEMENT

#6 RIGHT-TO-DIE

#7 HOSPICES

#8 RESPITE-CARE

#9 palliat*

#10 terminal* NEAR/6 care*

#11 terminal* NEAR/6 caring

#12 terminal* NEAR/6 ill*

#13 hospice*

#14 bereav*

#15 grief in Record Title OR grief in Abstract OR griev* in Record Title ORgriev* in Abstract

#16 right* NEAR/6 die*

#17 respite NEAR/6 care*

#18 respite NEAR/6 caring

#19 living NEXT will*

#20 advance* NEXT directive*

#21 advance* NEXT care NEXT plan

#22 ?end of life? NEAR/6 care

#23 ?end of life? NEAR/6 caring

#24 #1 OR #2 OR #3 OR #4 OR #5 OR #6 OR #7 OR #8 OR #9 OR #10 OR

 #11 OR #12 OR #13 OR #14 OR #15 OR #16 OR #17 OR #18 OR #19

 OR #20 OR #21 OR #22 OR #23 OR #24 OR #25 OR #26 OR #27 OR  #28 Or#29 OR #30 OR #31 OR #32 OR #33 OR #34

The search terms and strategy used to identify RCTs (displayed for the OVID Medline database) were as follows:

#1 randomized controlled trial.pt.

#2 controlled clinical trial.pt.

#3 randomized.ab.

#4 placebo.ab.

#5 drug therapy.fs.

#6 randomly.ab.

#7 trial.ab.

#8 groups.ab.

#9 1 or 2 or 3 or 4 or 5 or 6 or 7 or 8

#10 exp animals/ not humans.sh.

#11 9 not 10

**Appendix B: Potentially relevant studies**

Barkay O, Mosler P, Schmitt CM, Lehman GA, Frakes JT, Johanson JF, et al. Effect of endoscopic stenting of malignant bile duct obstruction on quality of life. Journal of clinical gastroenterology [Internet]. 2013; (6):[526-31 pp.].

Mok E, Lau KP, Lai T, Ching S. The meaning of life intervention for patients with advanced-stage cancer: development and pilot study. Oncology nursing forum [Internet]. 2012; (6):[E480-8 pp.].

Baczyk M, Milecki P, Pisarek M, Gut P, Antczak A, Hrab M. A prospective randomized trial: a comparison of the analgesic effect and toxicity of 153Sm radioisotope treatment in monotherapy and combined therapy including local external beam radiotherapy (EBRT) among metastatic castrate resistance prostate cancer (mCRPC) patients with painful bone metastases. Neoplasma [Internet]. 2013; (3):[328-33 pp.].

Zhang H, Liu L, Luo WY, Song YX, She W. Clinical analysis of vertebral sclerosis angioplasty and resection for the treatment of spinal metastases. [Chinese]. Chinese Journal of Cancer Prevention and Treatment. 2013 14 Mar;20(5):374-6. PubMed PMID: 2013665472. Chinese.

**Appendix C**

**Table C.1 Univariate meta-regression for the odds ratio of missing data at the primary end-point (no permutations) for all pre-defined covariates**

| Covariate | Odds ratio | P-value | Confidence intervals |
| --- | --- | --- | --- |
| Items of data requested (per doubling number of items) | 1.3 | **<0.001** | 1.1, 1.4 |
| Time to primary end-point (per doubling of days) | 1.1 | **0.001** | 1.06, 1.2 |
| Frequency of measuring primary outcome (per doubling of occasions) | 1.1 | 0.2 | 0.9, 1.4 |
| Total number of outcomes (per outcome/scale) | 1.02 | 0.6 | 1.0, 1.09 |
| Trial description  Pilot/phase2/ feasibility*  Phase 3  Full-scale RCT | 1.0  0.8  0.8 | 0.5  ―  0.3 | ―  0.3, 1.8  0.5, 1.2 |
| Trial design  Parallel*  Cross over  Cluster | 1.0  0.6  2.4 | 0.1  ―  0.2  0.09 | ―  0.3, 1.4  0.9, 6.6 |
| Sample size randomised | 1.0002 | 0.3 | 1.0, 1.0007 |
| Multicentre trial | 1.0 | 0.8 | 0.6, 1.5 |
| Funding  Government/private not for profit*  Private for profit  Not reported  No funding | 1.0  1.5  0.9  0.7 | 0.6  ―  0.4  0.6  0.4 | ―  0.6, 3.9  0.5, 1.4  0.3, 1.6 |
| Type of outcome  Symptom*  Psycho-spiritual  Quality-of-life  Other | 1.0  1.1  1.9  0.6 | **0.02**  ―  0.7  0.04  0.06 | ―  0.6, 2.2  1.02, 3.6  0.4, 1.01 |
| Type of intervention  Pharmacological*  Surgery/intervention^[[1]](#footnote-1)^  Psycho-spiritual  Service provision  Physical therapy  Other | 1.0  0.6  1.3  1.5  1.1  0.6 | 0.08  ―  0.1  0.5  0.2  0.8  0.1 | ―  0.3, 1.1  0.6, 2.4  0.8, 3.1  0.4, 3.0  0.4, 1.1 |
| Trial setting  Community/outpatient*  Inpatients  >1 setting  Not reported/unclear | 1.0  0.8  1.09  1.09 | 0.7  ―  0.5  0.7  0.3 | ―  0.5, 1.4  0.7, 1.8  0.7, 1.8 |
| Exclude patients based on age/performance status/extent of disease | 1.6 | **0.02** | 1.08, 2.5 |
| Used a method to minimise missing data | 1.1 | 0.6 | 0.7, 1.8 |
| Interview | 1.4 | 0.2 | 0.8, 2.2 |
| Diary | 0.8 | 0.6 | 0.4, 1.8 |
| Who collected the data  Research team alone*  Clinical +/- research team  Patient +/- research team  Not reported/unclear | 1.0  1.3  2.01  1.3 | 0.2  ―  0.4  0.06  0.4 | ―  0.7, 2.2  1.0, 4.2  0.7, 2.3 |
| Use of medical notes | 1.0 | 0.9 | 0.6, 1.6 |
| Phone participants to collect data | 1.2 | 0.6 | 0.6, 2.05 |
| Age | 1.0 | 0.6 | 1.0, 1.02 |
| Disease  Malignant*  Non-malignant  Mixed | 1.0  0.5  0.9 | 0.4  ―  0.2  0.8 | ―  0.2, 1.3  0.6, 1.6 |
| Performance status  PS 0/1*  2  3  4  Not reported/unclear | 1.0  1.1  1.03  0.7 | 0.8  ―  0.7  0.9  0.5 | ―  0.6, 2.2  0.5, 2.2  0.3, 1.8 |
| Random sequence generation  Low risk of bias*  High risk  Unclear | 1.0  1.6  1.09 | 0.8  ―  0.6  0.7 | ―  0.3, 9.2  0.7, 1.7 |
| Allocation concealment  Low risk of bias*  High risk  Unclear | 1.0  0.7  1.0 | 0.9  ―  0.6  1.0 | ―  0.2, 2.5  0.6, 1.6 |
| Blinding participants/personnel  Low risk of bias*  High risk  Unclear | 1.0  0.7  0.8 | 0.4  ―  0.2  0.5 | ―  0.4, 1.2  0.4, 1.5 |
| Blinding outcome assessment  Low risk of bias*  High risk  Unclear | 1.0  0.7  0.8 | 0.4  ―  0.2  0.5 | ―  0.4, 1.2  0.5, 1.5 |

- Reference category

**Appendix D: Univariate and multivariate meta-regression for differential rates of missing data**

Table D.1. Univariate meta-regression for differential rate of missing data

| Explanatory variable | Odds ratio | Standard error | P-value | 95% CI |
| --- | --- | --- | --- | --- |
| Intervention  Pharmacological*  Surgery  Psycho-social  Service provision  Physiotherapy/exercise  Communication | 1.0  1.4  1.2  1.1  1.4  0.9 | -  0.4  0.3  0.3  0.7  0.2 | 0.7  -  0.2  0.5  0.6  0.4  0.8 | -  0.8, 2.3  0.7, 1.9  0.7, 1.9  0.6, 3.5  0.6, 1.5 |
| Control  Other*  Standard care  Placebo | 1.0  0.8  0.7 | -  0.1  0.1 | 0.2  -  0.2  0.1 | -  0.5, 1.1  0.5, 1.07 |
| Participants blinded  Low risk of bias*  High risk of bias  Unclear risk of bias | 1.0  1.6  1.4 | -  0.3  0.3 | **0.09**  -  0.03  0.1 | -  1.05, 2.3  0.9, 2.04 |
| Items of data requested | 1.06 | 0.08 | 0.4 | 0.9, 1.2 |
| Time to primary end-point | 1.04 | 0.04 | 0.4 | 1.0, 1.1 |

*Reference category

Table D.2. Multivariate meta-regression for differential rate of missing data

| Explanatory variable | Odds ratio | Standard error | P-value | 95% CI |
| --- | --- | --- | --- | --- |
| Intervention  Pharmacological*  Surgery  Psycho-social  Service provision  Physiotherapy/exercise  Communication | 1.0  0.9  0.9  0.8  1.0  0.8 | -  0.3  0.3  0.3  0.5  0.3 | 1.0  -  0.8  0.6  0.6  0.9  0.4 | -  0.4, 1.9  0.4, 1.7  0.4, 1.6  0.4, 2.6  0.4, 1.5 |
| Control  Other*  Standard care  Placebo | 1.0  0.8  0.8 | -  0.2  0.3 | 0.6  -  0.4  0.5 | -  0.5, 1.3  0.4, 1.5 |
| Participants blinded  Low risk of bias*  High risk of bias  Unclear risk of bias | 1.0  1.6  1.4 | -  0.5  0.4 | 0.3  -  0.1  0.2 | -  0.9, 2.9  0.8, 2.5 |
| Items of data requested | 1.05 | 0.1 | 0.5 | 0.9, 1.3 |
| Time to primary end-point | 1.02 | 0.07 | 0.8 | 0.9, 1.2 |

* Reference category

1. Includes radiotherapy treatment

   *Reference category [↑](#footnote-ref-1)
